# Pregnancy, Contraception, and Menopause in Advanced Chronic Kidney Disease and Kidney Transplant

**DOI:** 10.1089/whr.2021.0053

**Published:** 2021-10-25

**Authors:** Virginia A. Dines, Vesna D. Garovic, Santosh Parashuram, Fernando G. Cosio, Andrea G. Kattah

**Affiliations:** ^1^ Division of Nephrology and Hypertension, Mayo Clinic, Rochester, Minnesota, USA.; ^2^ Department of Obstetrics and Gynecology, Mayo Clinic, Rochester, Minnesota, USA.

**Keywords:** chronic kidney disease, kidney transplant, contraception, menopause, pregnancy

## Abstract

***Background:*** Reproductive health is an essential part of the care of women with kidney disease. However, the self-reported patient experience of reproductive issues has been underexplored.

***Materials and Methods*:** We identified a cohort of women ages 18 to 44 at the time of kidney transplant from 1996 to 2014 at our 3-site program (*n* = 816). We sent each woman a survey on her reproductive lifespan, characterizing features from menarche to menopause.

***Results:*** We received survey responses from 190 patients (27%). One third of respondents reported amenorrhea before transplant, and 61.5% of these women reported resumption of menses post-transplant. The average age of menopause was 45.5 years, earlier than the general population (51.3 years). There were 204 pregnancies pretransplant and 52 pregnancies post-transplant. Pregnancies post-transplant were more likely to be complicated by preeclampsia, preterm delivery, and small for gestational age babies than pregnancies that occurred >5 years before transplant. Pregnancies <5 years before transplant were similar to post-transplant pregnancies with respect to complications. Forty-two percent of women were advised to avoid pregnancy after transplant, most often by a nephrology provider.

***Conclusions:*** In our cohort of kidney transplant recipients, women report increased pregnancy-related complications post-transplant and in the 5 years before transplant, compared with pregnancies that occurred greater than 5 years before transplant. They were often counseled to avoid pregnancy altogether. Women reported a younger age of menopause relative to the general population. This should be considered when counseling patients with chronic kidney disease regarding optimal pregnancy timing.

## Introduction

Reproductive health is an important aspect of medical care for women with chronic kidney disease (CKD) and kidney transplants. Women with CKD are considered a high-risk population in pregnancy, as there is a significantly increased risk of pregnancy-related complications and adverse maternal and fetal outcomes.^1–4^ Additionally, CKD affects fertility and patients may struggle to conceive.^5^ Post-transplant, women often have improved fertility; however, pregnancy post-transplant has additional risks, including potential exposure to teratogenic medications and increased risk of pregnancy complications and adverse outcomes compared with the general population.^6^ Women are often advised to wait until after a successful kidney transplant to pursue pregnancy, and major society guidelines recommend waiting to conceive 1–2 years post-transplant, depending on individual risk factors.^7^

For women in the advancing stages of CKD, planning a pregnancy can be further complicated by factors such as deterioration of kidney function, which could lead to the need for dialysis, and declining age-related fertility while awaiting a donor.^8^ As neonatal care has improved, as well as maternal and fetal outcomes of women on dialysis, whether to pursue pregnancy before or after transplant is less clear.^9,10^ Furthermore, data on the effects of pregnancy on kidney transplant outcomes have been limited and have shown mixed results, which complicates decision making regarding the optimal timing of pregnancy.^6,11–13^ Ideally, a multidisciplinary team, including obstetricians/gynecologists and nephrologists, can offer an individualized approach to each patient. Given the medical complexity and the multitude of personal factors involved in pursuing pregnancy, it is essential to better understand patients' experiences with reproductive issues.

The objective of this study was to better understand the reproductive health experiences of women who have had kidney transplants. Previous studies largely have focused on pregnancy-related clinical outcomes in CKD or post-transplant, or on individual components of reproductive health. However, several factors have been underexplored, including how many women have attempted pregnancy, how many have been advised against pursuing pregnancy, whether pregnancies post-transplant have more or fewer complications than pregnancies pretransplant, and whether a transplant could impact the timing of menopause, and therefore the “fertile window” after transplant. By answering the above questions, we hoped to identify gaps in knowledge for future study, as well as provide information to better guide counseling and care.

## Materials and Methods

We identified women ages 18 to 44 at the time of kidney transplant, from the years 1996 to 2014 at all three Mayo Clinic sites: Rochester, MN; Scottsdale, AZ; and Jacksonville, FL. We sent all women a survey at their most recent address. If women did not respond to the first survey, a second survey was sent. We followed up with a phone call after the second survey and resent a survey to women who wanted to participate. All survey responses were entered into a database. All subjects who declined to participate, either by phone or mail, were counted as refusals. This study was approved by the Institutional Review Board at Mayo Clinic.

### Transplant recipient characteristics

A manual chart review was performed using the 3-site transplant database. We recorded demographics at the time of transplant, including age and race, as well type of transplant (deceased vs. living donors), parity at time of transplant (any prior pregnancy vs. none), number of pregnancies before and after transplant, and body mass index (BMI).

### Survey

The survey was adapted from several existing surveys in collaboration with the Survey Research Center at Mayo Clinic, including the National Transplantation Pregnancy Registry,^14^ a previously validated survey on hypertension in pregnancy,^15^ and internal surveys used for clinical care in the Women's Health Clinic. The focus of the survey was general reproductive health, including pregnancy history before and after transplant, change in menstrual periods before and after transplant, use of contraception, and menopausal characteristics.

### Pregnancy history

We asked about the total number of pregnancies before and after kidney transplant and the outcomes of pregnancy, including the number of live births, stillbirths, neonatal deaths (death within the first 7 days of life), miscarriages, and elective abortions. We asked about whether each pregnancy lasting longer than 20 weeks had been planned, the gestational age at delivery, the number, sex and weights of the babies, the use of assisted reproductive technology, vaginal versus Cesarean delivery, and pregnancy complications. Pregnancy complications included the diagnosis of hypertension, preeclampsia, gestational diabetes, kidney infection, need for kidney biopsy, and rejection (for post-transplant pregnancies only). We also asked about the use of medications in pregnancy, focusing on immunosuppression. Responses regarding pregnancy outcomes were validated by manual chart review of a subset of patients who delivered at our institution and therefore had available pregnancy records.

We further inquired whether a woman had a desire for pregnancy in the future, had ever tried to get pregnant after transplant, whether she had been advised not to pursue pregnancy by a health care provider and about the use of contraception.

### Menarche and menopause

In addition to questions about pregnancy, we asked women about their ages at menarche and whether their periods had stopped before transplant. We also asked if their periods returned post-transplant, and if so, the timing of return. Finally, we inquired about their current menopausal status, history of oophorectomy and hysterectomy and their ages at last menstrual period.

### Statistical analyses

Data are presented as percentages for categorical variables and means (standard deviations) or median (interquartile range [IQR]) for continuous variables. We compared pregnancy characteristics and complications post-transplant to pregnancies pretransplant, according to the number of years before transplant (<5 years before transplant, 5–15 years before transplant, and >15 years before transplant). We compared demographic and transplant characteristics of respondents, nonrespondents, and refusals for the survey. We compared categorical variables using either Fisher's exact test or chi square test, as appropriate, and Student's *t*-test for continuous variables. Survey responses are presented as percentages, with the number of missing values noted. To determine clinical features associated with resumption of menses post-transplant, we used logistic regression models. For continuous predictors, we made receiver-operating characteristics curves and identified values with the highest sensitivity and specificity in predicting changes in menstrual cycles. Statistical analyses were conducted using JMP Pro version 14.1.0 (SAS, Cary, NC, USA).

## Results

There were 818 women ages 18 to 44 at the time of transplant at the three Mayo sites between January 1, 1996 and December 31, 2014. Of the 818 women in the cohort, 91 had died by the time of the mailing, 9 were currently living outside the United States, and 1 woman refused any mailings for research purposes. We were unable to send surveys to women living outside the United States. We mailed a total of 717 surveys and 190 women replied, 93 women refused to take the survey, and 18 more women were found to be deceased. The response rate was 27.2% (excluding women who were found to be deceased from total surveys sent).

Table 1 shows the demographic, transplant, and pregnancy characteristics of women who responded as well as those who did not respond. These data were obtained from chart review (Table 1). Women who responded were more likely to be white, transplanted between the years of 1996 to 2002 at the Rochester, MN site, and to have had a pregnancy after transplant than nonrespondents. Women who refused to complete the survey were older at the time of transplant, and less likely to have had a pregnancy after transplant than women who responded. There were no significant differences in transplant type (living vs. deceased), BMI at the time of transplant, or pregnancy before transplant among the three groups.

**Table 1.** Characteristics of Women Who Responded to the Survey, Women Who Refused to Participate, and Women Who Did Not Respond (Including Unclaimed Surveys)

Characteristic	Respondents (*n* = 190)	Refusals (*n* = 93)	Nonrespondents (*n* = 426)	*p*-Value; Respondents versus refusals	*p*-Value; Respondents versus nonrespondents
Age at transplant, mean (SD)	34.2 (7.7)	36.4 (6.3)	33.9 (7.0)	0.02^a^	0.68^a^
Year of transplant, *n* (%)				0.65^b^	0.007^c^
1996–2002	45 (23.7)	13 (14.0)	57 (13.4)		
2002–2008	59 (31.1)	32 (34.4)	156 (36.6)		
2008–2014	86 (45.3)	48 (51.6)	213 (50)		
Race, *n* (%)				0.16^c^	0.008^c^
White	148 (77.9)	66 (71.0)	280 (65.7)		
African American	12 (6.3)	8 (8.6)	64 (15.0)		
Asian or Asian Indian	7 (3.7)	3 (3.2)	21 (4.9)		
American Indian	4 (2.1)	2 (2.1)	20 (4.7)		
Other	19 (10.0)	14 (15.1)	41 (9.6)		
Site of transplant, *n* (%)				0.06^c^	<0.0001^c^
Rochester, MN	110 (57.9)	61 (66.6)	165 (38.8)		
Jacksonville, FL	28 (14.7)	18 (19.4)	101 (23.7)		
Scottsdale, AZ	52 (27.4)	14 (15.1)	160 (37.6)		
Transplant type, *n* (%)				0.27^c^	0.42^c^
Living donor	116 (61.1)	63 (67.7)	244 (57.6)		
Deceased donor	74 (39.0)	30 (32.3)	180 (42.4)		
Preemptive transplant, *n* (%)	25 (13.1)	14 (15.0)	54 (12.7)	0.56^b^	0.40^b^
BMI at transplant, mean (SD)	28.8 (0.6)	28.5 (0.9)	29.1 (0.5)	0.77^a^	0.77^a^
Ever pregnant before transplant, *n* (%)	76 (40)	31 (33.3)	184 (43.2)	0.30^c^	0.46^c^
Ever pregnant after transplant, *n* (%)	19 (10)	0 (0)	17 (4.0)	<0.0001^c^	0.005^c^

^a^ Student's *t*-test.

^b^ Fisher's exact test.

^c^ Chi square test.

BMI, body mass index; SD, standard deviation.

There were nine pregnancies with available records to review. We found that self-reported complications, birth weights, and gestational age at delivery were correct with the exception of two errors in the gestational age at delivery—one by 1 week and one by 10 weeks. Both deliveries remained preterm, but one reported a delivery at 20 weeks as opposed to 31 weeks, which could be due to misunderstanding the question or an error in writing 20 rather than 30, as an example.

### Pregnancy history

Table 2 shows the responses to questions about pregnancy. The majority of women reported being pregnant at some point (59.5%), with a median (range) of 2 (0–10) pregnancies overall. There were 17 women who reported one or more elective abortions before transplant, whereas only 1 woman reported an elective abortion post-transplant. There were 27 women who reported one or more miscarriages before transplant, and 12 women who reported one or more miscarriages after transplant.

**Table 2.** Responses to Questions About Pregnancy

Pregnancy questions	*N* = 190
Have you ever been pregnant? *n* (%)	
Yes	113 (59.4)
No	77 (40.5)
How many of the following have you had before kidney transplant? (median, range)	
Pregnancies	1 (0–8)
Miscarriages	0 (0–3)
Elective abortions	0 (0–3)
How many of the following have you had after kidney transplant? (median, range)	
Pregnancies	0 (0–9)
Miscarriages	0 (0–7)
Elective abortions	0 (0–1)
Are you planning to become pregnant in the future? *n* (%)	
Yes	11 (5.8)
No	157 (82.6)
Not sure/Had not thought about it	14 (7.4)
Missing	8 (4.2)
Since transplant, have you actively tried to become pregnant? *n* (%)	
Yes	27 (14.2)
No	157 (82.6)
Missing	6 (3.2)
Have you been advised against pregnancy by a health care provider? *n* (%)	
Yes	80 (42.1)
No	98 (51.6)
Missing	12 (6.3)
If yes, what type of provider recommended against pregnancy? *n* (%)	*N* = 80
Nephrologist or kidney transplant provider	42 (52.5)
Obstetrics–gynecology	13 (16.3)
Primary care or general internal medicine	5 (6.3)
Other	13 (16.3)
Missing	7 (8.8)

There was a total of 204 pretransplant pregnancies, the majority resulting in livebirths (67.1%). There were 141 pregnancies lasting longer than 20 weeks before transplant, with 4 stillbirths and 1 neonatal death. The majority of the women surveyed had pregnancies well before the time of transplant (median [IQR] 14 [8.5–18] years). Table 3 shows the characteristics of pregnancies lasting longer than 20 weeks' gestation. When looking only at pretransplant pregnancies, there was a trend toward younger maternal age (*p* < 0.0001, trend by analysis of variance [ANOVA]), later gestational age at delivery (*p* = 0.002), and higher birth weight (*p* < 0.001) the further the pregnancy was from the time of transplant.

**Table 3.** Comparison of Post-Transplant Pregnancy Outcomes on Pregnancies Lasting Longer Than 20 Weeks Gestation to Pretransplant Pregnancies at Increasing Intervals from Time of Transplant

	Post-transplant	Pretransplant						General U.S. population rates
		0–5 Years prior		5–15 Years prior		>15 Years prior		
	*N* = 30	*N* = 21	*p* ^a^	*N* = 66	*p* ^a^	*N* = 54	*p* ^a^	
Age at delivery (years)	32.7 (4.0)	28.2 (5.0)	0.007	27.1 (4.1)	<0.0001	20.9 (8.0)	<0.0001	
Planned pregnancy (yes)	26 (86.7%)	10 (47.6%)	<0.0001	37 (56.1%)	<0.0001	18 (33.3%)	<0.0001	
Stillbirth	0 (0%)	1 (4.8%)	0.41	1 (1.5%)	1.0	2 (3.7%)	0.54	0.59%^b^
Weeks at delivery	35.7 (2.3)	35.3 (4.7)	0.71	37.8 (2.4)	0.001	38.8 (4.2)	<0.0001	
Preterm delivery	21 (70%)	13 (65%)	0.76	18 (27.2%)	<0.0001	12 (22.2%)	<0.0001	10.23%^c^
Baby weight (g)	2502 (521)	2245 (859)	0.23	2952 (897)	0.003	3311 (816)	<0.0001	
Delivery type (% vaginal)	11 (36.7%)	10 (47.6%)	0.56	42 (65.6%)	0.008	35 (64.8%)	0.02	68.3%^c^
Hypertension	17 (56.7%)	14 (66.7%)	0.56	28 (42.4%)	0.27	23 (42.6%)	0.26	
Preeclampsia	15 (50%)	7 (33.3%)	0.27	18 (27.3%)	0.04	12 (22.2%)	0.01	
Gestational diabetes	1 (3.3%)	1 (4.8%)	1.0	1 (1.5%)	0.53	4 (7.4%)	0.65	

All data presented as *n* (%).

^a^ Fisher's exact test for categorical variables and Student's *t*-test for continuous variables. All in comparison to post-transplant pregnancies.

^b^ Data from National Vital Statistics Report 2020 on Fetal Death.^24^

^c^ Data from National Vital Statistics Report 2019 on Births.^25^

### Post-transplant pregnancies

A total of 27 women (14.2%) reported having actively tried to become pregnant after transplant, and 11 women (5.8%) reported actively trying to become pregnant at the time of the survey. Forty-two percent of women reported being advised against a pregnancy post-transplant. This recommendation was reported to have come from their nephrologists or kidney transplant providers more than half the time (52.5%). The other half of respondents reported this advice came from obstetricians and gynecologists (16.3%), primary care providers (6.3%), and others (16.3%). Table 4 outlines the reasons respondents reported for being advised against pregnancy. The most common reason given was concern for teratogenicity of immunosuppressant medication or risk of rejection from altering medications (25.9%). There were several women who listed concern for loss of kidney function (17.2%) and concern for exacerbation or complication related to primary kidney disease (14.8%). Eight (9.8%) women reported being given no reason, or the only reason given was that they had a transplant.

**Table 4.** Reason for Being Advised Against Pregnancy

No. of women advised against pregnancy	*N* = 81
Reasons given for being advised against pregnancy,^a^ *n* (%)	
No reason offered or only reason having had a transplant	8 (9.8)
Teratogenicity of immunosuppressant medications or concern that adjusting medications would lead to rejection	21 (25.9)
Risk of complications or exacerbation of primary disease, causing kidney failure	12 (14.8)
Risk of loss of kidney function	14 (17.2)
Advanced age or postmenopausal	5 (6.2)
Personal history of pregnancy related complications	4 (4.9)
Uncontrolled or difficult to control hypertension	2 (2.5)
Too early in post-transplant course	2 (2.5)
General concern regarding health of mother and/or fetus	16 (19.8)
Previous tubal ligation or uterine ablation	2 (2.5)
Health concern unrelated to kidney disease or transplant status	7 (8.6)

^a^ Nos. add up to greater than 81 as women could list more than 1 reason.

There were 52 different pregnancies in 25 unique women at a median (IQR) of 6 (3.75–10.25) years post-transplant. The majority of pregnancies resulted in livebirths (*n* = 30, 57.7%), with the rest being miscarriages (*n* = 22, 42.3%). There were two women whose pregnancies accounted for 9 of the 22 (40.9%) miscarriages.

For the 30 pregnancies that lasted longer than 20 weeks, the pregnancy was planned in 86.7% of cases. There were two twin gestations, one of which was conceived with assisted reproductive technology. Only two women reported using assisted reproductive technology. The median (IQR) gestational weeks at delivery was 36 (34.8–37), and 70% of deliveries were preterm. The median (IQR) gestational weight at delivery was 2589 (2098–2863) g. The majority of deliveries were by C-section (63.3%) as opposed to vaginal delivery (36.7%).

The majority of pregnancies were complicated by hypertension in pregnancy (56.7%), although only 50% reported that they were diagnosed with preeclampsia, specifically. One woman reported each of the following complications: gestational diabetes, rejection episode diagnosed by kidney biopsy, and placental abruption. There were four women who reported rising creatinine in pregnancy, only two of which also reported preeclampsia in that pregnancy. In terms of immunosuppression, women took prednisone (76.7%), tacrolimus (90%), and azathioprine (76.7%). Three women reported taking mycophenolate mofetil in pregnancy at some point. Two of these were pregnancies earlier in the cohort period (1998 and 2002). Five women reported being on a beta-blocker for chronic hypertension in pregnancy.

### Comparing pre- and post-transplant pregnancies

Table 3 shows the characteristics of pregnancies lasting longer than 20 weeks post-transplant and pretransplant at 3 different time points: 0–5 years before transplant, 5–15 years before transplant, and >15 years before transplant. Pregnancy outcomes were based on patient-reported information *via* survey responses. In pregnancies post-transplant, recipients were more likely to be older at the time of delivery, with a mean age of 32.7 years. Post-transplant pregnancies were also more likely to be planned (86.7%) relative to pregnancies pretransplant (*p* < 0.0001 for all time points). Deliveries were more likely to be preterm (70%), with an average delivery at 35.7 weeks relative to pregnancies 5–15 years pretransplant (*p* = 0.001) and >15 years pretransplant (*p* < 0.0001), but there was no difference in pregnancies 0–5 years pretransplant. Babies born from post-transplant pregnancies were also more likely to be smaller and born *via* C-section than those born from pregnancies >5 years pretransplant. There was no difference in rates of hypertension or gestational diabetes between pregnancies pre- and post-transplant. Pregnancies post-transplant were more likely to be complicated by preeclampsia than those pregnancies >5 years pretransplant; however, there was no difference relative to pregnancies 0–5 years pretransplant. Rates of stillbirth were very low in all time periods.

### Contraception

Only 95 women (50%) reported having used contraception at any point after transplantation, and 68 women (35.8%) were using contraception at the time of survey response. Contraception was used for a median (range) of 10 (5–16.8) years. Of the 68 women using contraception at the time of survey response, 45% were using permanent sterilization, including bilateral tubal ligation (20.6%) and vasectomy (25%). Long-acting reversible contraceptives were also commonly reported, including an intrauterine device with levonorgestrel in 12 (17.6%) women. Less effective methods, such as condoms/barrier and natural family planning methods, were reported by 14 (20.6%) and 3 (2.9%) women, respectively.

### Menopausal characteristics

Details of the responses to questions regarding menopause and menstruation are shown in Table 5. Thirty-seven percent of respondents were premenopausal, 14.2% were perimenopausal, 27.9% were postmenopausal, and 17.4% of respondents were unsure of their menopausal status. In women who were postmenopausal, the average age of their last period was 44.5 years, with an IQR of 36–49 years. Twenty-seven women (14.2%) had a hysterectomy, 14 before transplant and 13 post-transplant. Eleven women (5.8%) reported a previous oophorectomy, with five before transplant and six post-transplant.

**Table 5.** Responses to Questions About Menstrual Cycles and Menopause

Menstrual cycles and menopause	*N* = 190
At what age did menstrual periods start? median (IQR)	13 (12–14)
What is your current menopausal status? *n* (%)	
Premenopausal	67 (31.6)
Perimenopause	27 (14.2)
Menopausal	53 (27.9)
Not sure	33 (17.4)
Missing	10 (5.3)
If you are menopausal, at what age did you have your last period? median (IQR)	44.5 (36–49) years
Did you have a hysterectomy? *n* (%)	
Yes, before transplant	14 (7.4)
Yes, after transplant	13 (6.8)
No	158 (83.1)
Missing	5 (2.6)
Did you have an oophorectomy? *n* (%)	
Yes, before transplant	5 (2.6)
Yes, after transplant	6 (3.2)
No	173 (91.1)
Missing	6 (3.2)
Did your period stop before transplant? *n* (%)	
Yes	63 (33.1)
No	117 (61.6)
Missing	10 (5.7)
If yes, did your period start after transplant? *n* (%)	*N* = 63
Yes	40 (63.5)
No^a^	25 (39.7)
How long after transplant did your periods restart? median (IQR)	3 (2–6.5) months

^a^ Ten had either hysterectomy, oophorectomy, or both.

IQR, interquartile range.

Approximately one-third of patients (34.2%) reported that their periods had stopped before transplant. Of these patients, 61.5% had their periods return and 38.5% reported that their periods did not return; however 10 of these patients had a hysterectomy, oophorectomy, or both, and these patients were excluded from the logistic regression models. Age at transplant was a significant predictor of menses returning post-transplant (odds ratio [OR] 0.81, 95% confidence interval [CI] 0.70–0.93 per year), with age of 39 having the highest sensitivity (85%) and specificity (69%) for menses not resuming post-transplant. BMI at transplant was also a significant predictor for resumption of menses (OR 0.91, 95% CI 0.84–0.98), with BMI of 26.0 at the time of transplant having the highest sensitivity (58%) and specificity (92%). Deceased versus living donor transplant, pregnancy before transplant, and transplant site were not significantly different between women who resumed menses and those who did not. The average time from transplant to resumption of menstrual periods was 3 months (range of 2–6.5 months).

## Discussion

In this study, we have analyzed the reproductive histories and experiences of 190 patients with a previous kidney transplant using a patient survey. Previous studies have focused more specifically on pregnancy and kidney function before and after transplant, while not exploring other issues related to reproduction.^16^ An understanding of pregnancy outcomes and fertility pre- and post-transplant is important for many women with kidney disease who may wish to pursue pregnancy, and this study offers important insights into some of the challenges faced by women with kidney disease. Outcomes such as preeclampsia and preterm delivery, in particular, were as common in pregnancies within 5 years pretransplant as they were in pregnancies post-transplant, raising important questions about pregnancy timing. Women post-transplant were more likely to have pregnancies at an older age, which carries increased risk of pregnancy complications. Furthermore, the earlier reported age at menopause suggests that the fertile window post-transplant may be shortened in certain women. We found that the majority of pregnancies post-transplant were planned, but that there is room to improve use of contraception post-transplant. Overall, these findings reflect the complexity involved in counseling women with advanced CKD and kidney transplants on reproductive health.

In our cohort, there were important differences in pregnancy outcomes relative to the proximity to kidney transplant. Post-transplant pregnancies, which typically occurred within 4–10 years post-transplant, did have not significantly different outcomes than pregnancies that occurred 0–5 years pretransplant. The high rate of complications in the more immediate pretransplant period likely reflects the presence of advanced CKD, whereas pregnancies >5 years pretransplant likely occurred in the setting of milder, if any, CKD. A meta-analysis examining pregnancy outcomes in CKD patients, which included 10 studies on preeclampsia risk, showed an OR of 10.36 (95% CI 6.28–17.09) for risk of preeclampsia in patients with CKD relative to patients without CKD. Additionally, patients with CKD have been shown to have increased risk of preterm delivery and lower birth weight babies, as well as increased C-section rates.^2^ Interestingly, we did not find a significant difference in outcomes between post-transplant pregnancy and pregnancies <5 years before transplant, presumably when a woman has established CKD. To our knowledge, this is the first study that compares pregnancy outcomes post-transplant relative to pretransplant at multiple time points in a single cohort. However, we do not have data regarding the stage of kidney disease or graft function at the time of pregnancies before and after transplant, which are also important factors.

The rate of preeclampsia reported in post-transplant pregnancies was higher than previously reported in a meta-analysis.^6^ However, consistent with previous studies, the rate is well above the average rate in the general nontransplant population in the United States (3.3%).^17^ Our study is consistent with previous studies in that risk of preterm delivery, having a small-for-gestational-age baby, and C-section, were all increased post-transplant relative to pregnancies at least 5 years before transplant.^6^ Rates of these complications in our study were similar to those described in previous cohort studies.^18^ The majority of post-transplant pregnancies reported resulted in live births; however, the miscarriage rate was 42.3%, which is greater than the risk in the general population of 17.1%, although two women accounted for nearly half of these miscarriages. Our results are also likely impacted by nonresponse bias in that we found that women who replied were more likely to have had a post-transplant pregnancy by chart review.

We found that the many patients had been advised against pregnancy by their physicians, most commonly their transplant physicians or nephrologists, although also frequently by obstetricians or primary care providers. Nephrologists frequently report low confidence in providing contraception counseling and report a lack of training in counseling women regarding reproductive health,^19^ and thus this raises an important opportunity for multispecialty collaboration. Many of the women reported being advised against pregnancy due to a general concern for their health, while in other cases it was more specifically due to concern for declining kidney function. Pregnancy certainly poses a unique risk to kidney transplant patients. In previous studies, ∼25% of patients have experienced a serious complication of pregnancy from a post-transplant pregnancy.^6^ However, several patients reported not being provided the reason or not understanding the reason for avoiding pregnancy, suggesting that providers may not be fully counseling women on their reproductive options. Despite the importance of contraceptive counseling in this patient population, only 50% of patients surveyed reported using contraception at any point post-transplant, although highly effective methods, such as sterilization and long-acting reversible contraceptives, were reported with high frequency, and 86.7% of post-transplant pregnancies were planned. This represents an area for clinical improvement, as patients would benefit from multispecialty collaboration and counseling on contraception and reproductive health. Individualized counseling is of utmost importance as different patients may have different perspectives on the risks and benefits of pursuing pregnancy and it can be difficult to quantify different priorities. This study did not address the individual's relative prioritization of fertility to transplant health and graft function.

Reproductive counseling is also essential due to risk of teratogenicity associated with mycophenolate mofetil.^20^ In our study, the majority of women were on prednisone, azathioprine, and tacrolimus during their post-transplant pregnancies. These are all known to be safe immunosuppressant medications in pregnancy.^1,21^ Three women were on mycophenolate mofetil during their pregnancies at some point, two of them early in the cohort (1998 and 2002). The most common reported reason for being advised against pregnancy was concern regarding teratogenicity of medications or need for medication changes, indicating that patients are being counseled on teratogenicity.

We were able to better understand the rates of return of menstruation after kidney transplant and timing of menopause post-transplant. Notably, our patients reported that their average age of menopause was 45.5 years, which is well below the average age of menopause in U.S. women (51.3 years).^22^ While previous studies have reported earlier menopause in women on dialysis due to disruptions in the hypothalamic–pituitary–gonadal axis, this finding in women who have generally had a restoration in kidney function post-transplant is unexpected.^8^ In women with CKD and end stage kidney disease (ESKD), it is thought that alterations in gonadotropin releasing hormone secretion leads to losses of normal follicle stimulating hormone (FSH) and luteinizing hormone surges, which in turn, cause low estradiol and menstrual irregularities, including amenorrhea. Hyperprolactinemia is also common in ESKD patients and may contribute to abnormal reproductive hormone levels.^5^ In patients with CKD, it remains unclear as to what point kidney function deterioration affects the hypothalamic–pituitary axis. Women with amenorrhea on dialysis have been shown to have lower FSH levels than those consistent with menopausal levels, and thus they are considered to be in a pseudomenopausal state, as often kidney transplant leads to resumption of menstruation.^5,8,23^ We saw this in our survey results, as approximately one-third of surveyed patients reported their periods stopped before transplant, and of those patients, 61.5% had their periods resume post-transplant. Older age at transplant and higher BMI were associated with lower odds of resumption of menstrual cycles after transplant. A better understanding as to why some women do not resume menstrual cycles post-transplant should be a focus of future study.

Our study has several strengths. We were able to include patients from all three Mayo Clinic sites to develop a large and diverse cohort. Using the survey, we were able to review the reproductive history of a large patient population. We were able to assess patient-reported outcomes and gain patient perspectives on reproductive decisions such as contraceptive use and pregnancy counseling. We were able to capture additional pregnancies and pregnancy outcomes using survey methodology that were not available by chart review alone. This is evidenced by the difference in total pregnancies before and after transplant identified in Table 1 by chart review and those in Table 2 by survey responses.

Our main limitation was the response rate of 27%. Additionally, survey data depend on self-reporting, and thus may be subject to recall bias. There were some significant differences between survey respondents and nonrespondents, including age, race, and rates of post-transplant pregnancy. Thus, our results may not fully reflect transplant patients in general. Additionally, we were unable to fully assess the impacts of potential pregnancy without a comprehensive chart review. This was a retrospective study; thus, results should be interpreted with caution. Future work should focus on expanding the patient population to a more diverse cohort and a prospective study. This study offers important insights in the timing of pregnancy and may help clinicians and patients in discussing optimal pregnancy timing.

## Conclusion

The results of this study show an increased risk of pregnancy-related complications in post-transplant pregnancies relative to pregnancies greater than 5 years before kidney transplant. Outcomes in pregnancies less than 5 years before kidney transplant were similar to those in post-transplant pregnancies. Women in our cohort also reported a younger age of menopause relative to the general population and many women experience amenorrhea related to CKD. These findings offer insights into optimal timing of pregnancy and provide additional information for patients and clinicians.

## Data Availability

The data underlying this article are available in the article. The survey is available upon request.

## Abbreviations Used

BMI: body mass index
CI: confidence interval
CKD: chronic kidney disease
ESKD: end stage kidney disease
FSH: follicle stimulating hormone
IQR: interquartile range
OR: odds ratio
